# Proteomic Analysis of the Murine Liver Response to Oral Exposure to Aflatoxin B1 and Ochratoxin A: The Protective Role to Bioactive Compounds

**DOI:** 10.3390/toxins17010029

**Published:** 2025-01-09

**Authors:** Silvia Trombetti, Alessandra Cimbalo, Michela Grosso, Pilar Vila-Donat, Jordi Mañes, Lara Manyes

**Affiliations:** 1 Department of Molecular Medicine and Medical Biotechnology, University of Naples Federico II, Via Pansini 5, 80131 Naples, Italy; silvia.trombetti@unina.it (S.T.); michela.grosso@unina.it (M.G.); 2 Biotech Agrifood, Faculty of Pharmacy and Food Sciences, Universitat de València, Avda. Vicent Andrés Estellés s/n, 46100 Burjassot, Spain; alessandra.cimbalo@uv.es (A.C.); jordi.manes@uv.es (J.M.); lara.manyes@uv.es (L.M.)

**Keywords:** proteomics, mycotoxin, bioactive compounds, in vivo, LC-MS/MS-QTOF

## Abstract

Aflatoxin B1 (AFB1) and Ochratoxin A (OTA) are considered the most important mycotoxins in terms of food safety. The aim of this study was to evaluate the hepatotoxicity of AFB1 and OTA exposure in Wistar rats and to assess the beneficial effect of fermented whey (FW) and pumpkin (P) as functional ingredients through a proteomic approach. For the experimental procedures, rats were fed AFB1 and OTA individually or in combination, with the addition of FW or a FW-P mixture during 28 days. For proteomics analysis, peptides were separated using a LC-MS/MS-QTOF system and differentially expressed proteins (DEPs) were statistically filtered (*p* < 0.05) distinguishing males from females. Gene ontology visualization allowed the identification of proteins involved in important biological processes such as the response to xenobiotic stimuli and liver development. Likewise, KEGG pathway analysis reported the metabolic routes as the most affected, followed by carbon metabolism and biosynthesis of amino acids. Overall, the results highlighted a strong downregulation of DEPs in the presence of AFB1 and OTA individually but not with the mixture of both, suggesting a synergistic effect. However, FW and P have helped in the mitigation of processes triggered by mycotoxins.

## 1. Introduction

Aflatoxin B1 (AFB1) and Ochratoxin A (OTA) are two major mycotoxins which contaminate a wide range of food commodities, especially cereals and derived products, representing a serious concern for human and animal health [1]. These toxic compounds are secondary metabolites produced by filamentous fungi that belong primarily to *Aspergillus* and *Penicillium* species and grow on crops under conditions of improper storage and humidity. Within the two, AFB1 is the most potent hepatotoxic and carcinogenic member of the aflatoxin family whereas OTA is a nephrotoxic and immunosuppressive compound. Due to their severe toxicity, AFB1 and OTA are classified by the International Agency for Research on Cancer (IARC) as a Group 1 carcinogen (carcinogenic to human) and Group 2B carcinogen (possibly carcinogenic to humans), respectively [2].

Over the years, it has been demonstrated that these toxins are widespread in various species of cereals such as wheat, maize, barley, and rice and can occur during pre-harvest, post-harvest, or storage stages. In fact, diverse climatic factors including high humidity and temperature markedly increase mold growth, leading to its contamination. Therefore, the consumption of contaminated cereals and their derivatives, such as flour, breakfast cereals, and processed food products, represents a significant health hazard, since these toxins are stable even under cooking and processing conditions [3]. With regard to their toxicological effects on humans, it has been demonstrated that long-term exposure to AFB1 and OTA can lead to several health disorders, among which is the onset of liver damage and cancer [4,5].

Accordingly, the liver plays a central role in AFB1 and OTA metabolism. In fact, it has been demonstrated that AFB1 is primarily bioactivated by hepatic microsomal phase I cytochrome P450 enzymes, which are able to convert it into its electrophilic reactive epoxide form (AFBO). Consequently, this metabolite form adducts to DNA and proteins, causing mutations and promoting liver carcinogenesis. However, AFBO can also be metabolized by phase II detoxifying enzymes, leading to its degradation and elimination [6]. Similarly, OTA is biotransformed in the liver by phase I and II enzymes, but nonetheless, it is not the sole organ to metabolize this toxin [7].

Given the broad presence of AFB1 and OTA in food commodities and especially the difficulty of their elimination, the research has focused on the possibility of employing substances capable of modifying their metabolism and reducing their bioaccumulation. Lactic acid bacteria (LAB), for instance, are able to increase the quality of food matrices by producing a rapid fermentation and synthesizing a wide range of beneficial molecules [8]. Among them, organic acids reduce the pH of the substrates, preventing growth of undesirable microorganisms such as mycotoxigenic fungi [9]. In addition to probiotics, plant-based foods like pumpkin (P) (*Cucurbita* spp.) are rich in antioxidants, making them effective in combating oxidative stress. In fact, they contain high levels of bioactive compounds such as carotenoids, vitamin C, and phenolic compounds, which contribute to its strong antioxidant capacity [10]. Moreover, these compounds help reduce chronic inflammation, a factor in diseases like cancer and cardiovascular conditions, making pumpkin a valuable dietary component for mitigating the harmful effects of environmental toxins. In this study, fermented whey (FW) and P as functional ingredients were used either individually or in combination to replicate a realistic scenario in the Mediterranean diet. Moreover, the intake of a single functional compound is implausible as natural foods always contain numerous bioactive compounds [11].

From the perspective of the food industry, the production of 1 kg of cheese generates about 9 L of whey, almost half of which is disposed of as waste. This disposal, often untreated, poses significant environmental problems [12]. Considering that whey offers a promising solution to counter the harmful effects of mycotoxins, harnessing the bioactive components of fermented whey not only solves whey disposal problems but also provides a sustainable way to mitigate the associated risks, turning an environmental liability into a valuable resource [13]. Moreover, several studies have focused on its hepatoprotective effects against acute or chronic toxicity induced by xenobiotics [14,15,16,17].

It is also important to emphasize that the use of proteomics has proven to be a valuable tool for deepening the understanding of the mechanisms of action that cause hepatotoxicity, since it enables the identification and quantification of specific proteins associated with toxic responses and protective pathways, which are derived from FW and P interventions. Furthermore, it is a key element for identifying important biomarkers related to various liver diseases and even cancer [18,19].

In light of this, the aim of the present study was to investigate the advantageous role of goat milk FW and P as functional ingredients in safeguarding the sub-chronic hepatotoxic effects of AFB1 and OTA in male and female rats through a proteomics approach.

## 2. Results and Discussion

### 2.1. Identification and Quantification of Proteins

Gel-free shotgun proteomics analysis of rat liver was initiated by identifying peptides features through Spectrum Mill MS Proteomics Workbench Package Rev BI.07.09 (Agilent Technologies, Santa Clara, CA, USA). Thereafter, the proteins with different abundances between groups were statistically filtered by Mass Profiler Professional 15.0 version software (Agilent Technologies, Santa Clara, CA, USA) through an unpaired *t*-test (*p* < 0.05) distinguishing males from females of each experimental group. More specifically, each group exposed to single or combined mycotoxins was compared with its counterpart supplemented with functional ingredients, once with FW and once with FW + P, in order to identify the DEPs involved.

**Figure 1:**
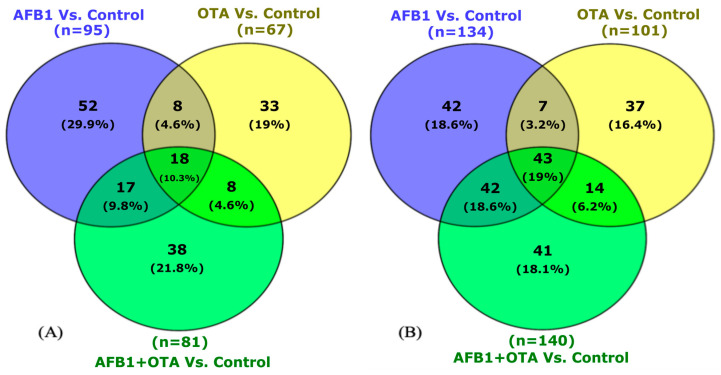
Venn diagram representation of common DEPs for male (**A**) and female (**B**) rats exposed to mycotoxins versus the control. *p* < 0.05 were significantly different from the control.

In male rats exposed to mycotoxins (Figure 1A), 95 proteins were differentially expressed in the AFB1 group compared to the male control group, 67 with OTA versus the control group, and 81 with the combination (AFB1 + OTA vs. control). In females (Figure 1B), more DEPs were observed for each comparison: 134 were identified with AFB1, 101 with OTA, and 140 with the combination.

**Figure 2:**
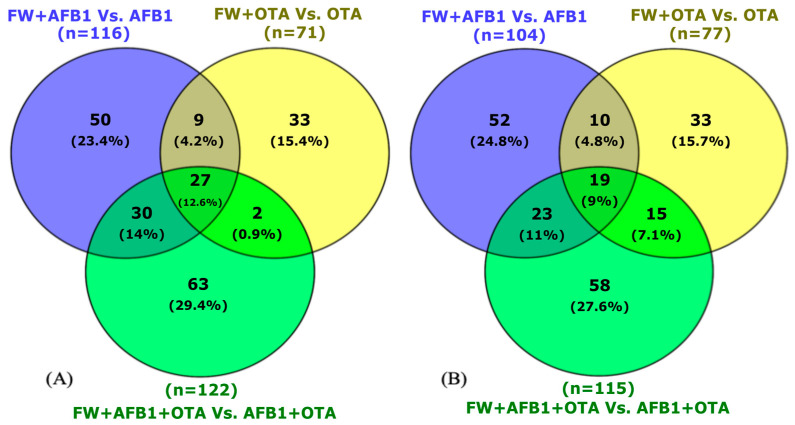
Venn diagram representation of common DEPs for male (**A**) and female (**B**) rats exposed to FW and mycotoxins versus the corresponding mycotoxin. *p* < 0.05 were significantly different from mycotoxins group.

In male rats exposed to FW (Figure 2A), 116 proteins were differentially expressed in the FW + AFB1 group with respect to the one with only AFB1, 71 with OTA versus OTA group, and 122 with the combination (FW + AFB1 + OTA vs. AFB1 + OTA). In females (Figure 2B), a similar scenario is observed: 104 were identified with AFB1, 77 with OTA, and 115 with the combination.

**Figure 3:**
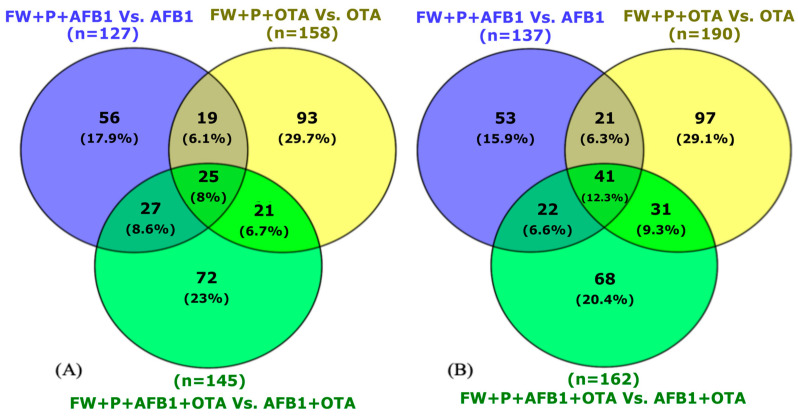
Venn diagram representation of common DEPs for male (**A**) and female (**B**) rats exposed to FW + P and mycotoxins versus the corresponding mycotoxin. *p* < 0.05 were significantly different from mycotoxins group.

In the presence of FW and P (Figure 3), the DEPs figure was higher than single FW when mycotoxins were administered individually, reporting a number of 127 proteins for males (Figure 3A) and 137 for females (Figure 3B) with AFB1 compared to 158 and 190 for males and females exposed to OTA, respectively. However, in the presence of both mycotoxins, the number decreased to 145 for males and 162 for females.

### 2.2. Gene Ontology of Differentially Expressed Proteins

Functional annotation of the differentially expressed proteins (DEPs) was performed using the DAVID database [20] in order to identify the most significant biological processes (BPs) and molecular functions (MFs) involved in DEPs found in each comparison. The feed exposure to AFB1 affected hepatic metabolism. Compared to control, it altered the expression of urea cycle, glycolysis and gluconeogenesis, and amino acid biosynthesis proteins, which is in line with the results found by Sun et al. (2019) that reported an upregulation of proteins involved in cancer-related pathways of metabolism, amino acid biosynthesis, and chemical carcinogenesis [21]. Furthermore, it caused oxidative stress. These results were mostly observed in females in which, besides Hsp70 overregulation, Gpx1 and Sod1 downregulated expression was identified. Exposure to OTA feed causing an altered response to oxidative stress was evident in both sexes. The effects were similar, but the proteins involved were different, except for Prdx1 which resulted downregulation in both sexes. Its downregulation has been associated with the activation of the PI3K/AKT pathway and therefore the promotion of cancer [22]. In addition, ATP synthase F1 subunit beta (Atp5f1b), an important protein for hepatic mitochondrial function, was significantly downregulated as much in males as in females. Several studies demonstrated that the reduction in its expression exacerbates mitochondrial dysfunction and oxidative stress [23]. When rats were fed with AFB1 + OTA, changes occurred in the expression of proteins involved in metabolism such as the urea cycle, glycolysis/gluconeogenesis, and amino acid biosynthesis as in the AFB1 case. In addition, oxidative effects were observed. Specifically, in females, several antioxidant enzymes (Gta1, Gstm1, Sod1, and Cat) were downregulated. Along with these findings, the downregulation of these enzymes has been associated with the onset of diverse cancers [24,25]. Only after exposure to both mycotoxins did a reduction in the expression of structural chromatin constituents occur. Reduced expression of these components could have a negative effect on the maintenance of genome integrity.

**Figure 4:**
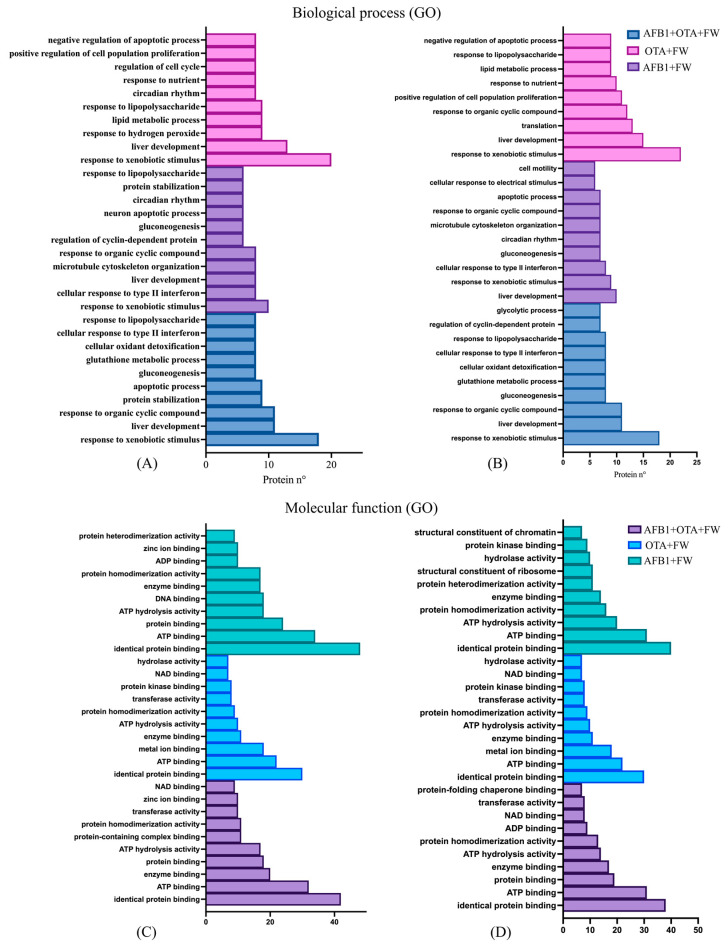
Gene ontology (GO) functional annotation of differentially expressed proteins for biological processes and molecular functions of male (**A**,**C**) and female (**B**,**D**) rats exposed to FW + AFB1, FW + OTA, and FW + AFB1 + OTA compared with respective mycotoxins without functional ingredient.

After exposure to mycotoxins and the individual functional ingredient (FW), the response to xenobiotic stimulus emerged as the most significant BP in both males (*n* = 18 to 20) and females (*n* = 9 to 17) (Figure 4A,B).

**Figure 5:**
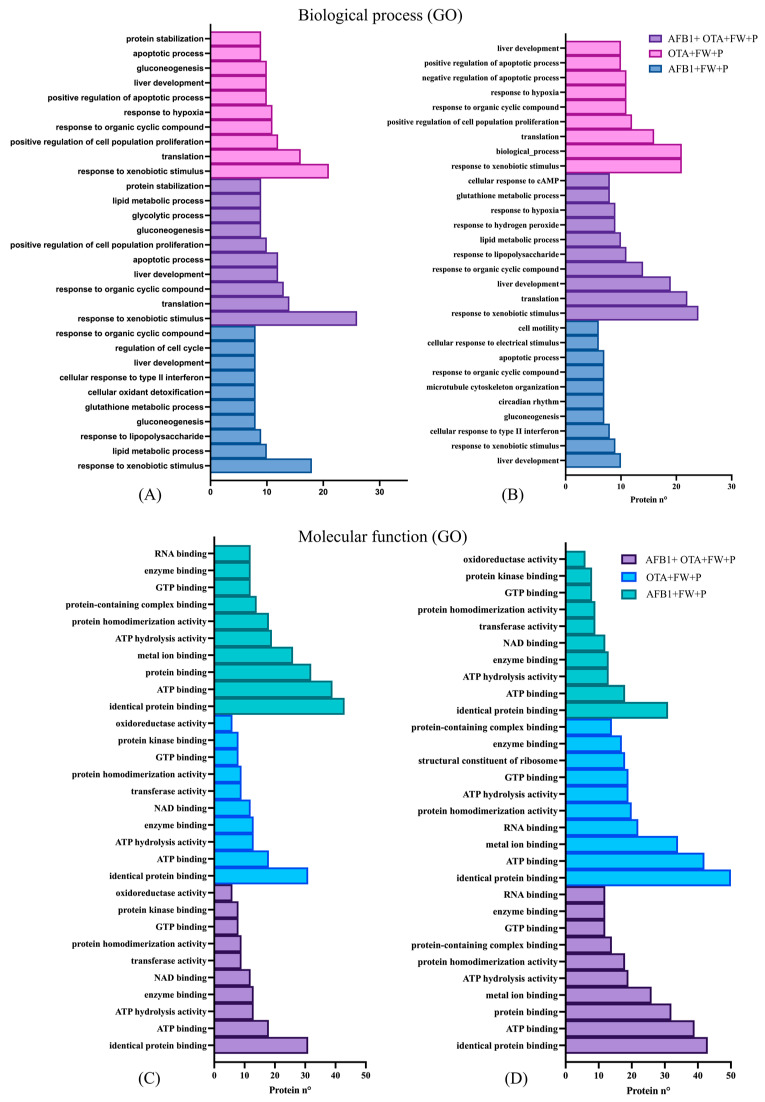
Gene ontology (GO) functional annotation of differentially expressed proteins for biological processes and molecular functions of male (**A**,**C**) and female (**B**,**D**) rats exposed to FW + P + AFB1, FW + P + OTA, and FW + P + AFB1 + OTA compared with respective mycotoxins without functional ingredients.

Likewise, similar findings were observed when rats were exposed to both functional ingredients (Figure 5). The response to xenobiotic stimulus was the most common biological process in male (n = 18–20) and female rats (n = 9), though it was more pronounced in males.

According to that, it is well known that liver plays a crucial role in metabolizing and detoxifying xenobiotics via phase I and phase II enzymes, and many of the proteins involved are key players in the detoxification pathways activated by AFB1 and OTA exposure. In fact, the upregulation of oxidative proteins suggests higher oxidative stress in the liver, which could indeed be part of a positive feedback mechanism that the liver cells use to maintain homeostasis. While these parameters may not directly reflect hepatotoxicity, they offer valuable insight into the liver’s adaptive response to stress [26]. Among the proteins affected, the most significantly altered were the mitochondrial enzyme involved in ketogenesis Hmgcs2, glutathione S-transferases (Gsta1, Gstm1, Mgst1) which conjugate toxic metabolites with glutathione to facilitate their excretion, and oxidative stress biomarkers superoxide dismutase 1 (Sod1) and catalase (Cat). Moreover, heat shock proteins such as Hspa8 and Hspd1 which protect cells from stress-induced damage were upregulated with the combination of toxins (Log Fold Change (FC) > 2) but not with the individual exposure (LogFC < −2). Additionally, enzymes implicated in energy metabolism and cellular repair (Aldh9a1, Adcy1) were strongly downregulated in the combined exposure (LogFC < −1.80) but not in the single ones, suggesting a synergistic effect of the toxins. According to that, previous studies confirmed the hepatotoxic modulation of xenobiotics metabolizing enzymes in the presence of AFB1, but at the same time, the capacity of coffee extracts to activate detoxifying enzymes for its degradation was demonstrated [27]. Likewise, the degradation of AFB1 was recently proven by employing diverse bacteria species, as well as different waste products containing high amounts of phenolic compounds [28,29]. Moreover, the modulation of the xenobiotic transformation system induced by OTA was involved in hepatic metabolism processes in vitro and in vivo [30,31]. However, as in this case, it has been demonstrated that plant extracts and their bioactive compounds may act by inducing xenobiotic detoxification and biotransformation pathways [32].

The following foremost BP was related to liver development with both functional ingredients and in the two sexes, reporting a number of 10 to 13 findings for males (Figure 4A and Figure 5A) and 8 to 10 for females (Figure 4B and Figure 5B). This biological mechanism is essential for growth, differentiation, and maturation of the liver and is tightly regulated by various signaling pathways and proteins that control cellular functions such as proliferation, differentiation, and metabolic adaptation. In the context of mycotoxin exposure, proteins such as Atp5f1b, UDP glucuronosyltransferase family 1 member A6 (Ugt1a6), adenylate kinase 2 (Ak2), and aldehyde dehydrogenase 9 family member A1 (Aldh9a1) and ornithine transcarbamylase (Otc) were downregulated when the rats were exposed to mycotoxins individually. Accordingly, diverse studies have reported the healthful effect of bioactive components contained in food in the increase in cellular antioxidant defense systems at the hepatic level [33]. Therefore, these results indicate that the combined action of these bioactive ingredients may actively participate in favorable mitigation processes. However, when both AFB1 and OTA were administered together, the expression of these proteins was notably increased, suggesting an adaptive response by the liver to counteract the toxic effects and promote recovery. Additionally, BPs related to glutathione metabolism, apoptotic processes, gluconeogenesis, response to nutrients, and circadian rhythm were also affected, but in a lower manner. In terms of MFs (Figure 4C,D and Figure 5C,D), identical protein binding was the most enriched function in both sexes (n = 30 to 48), followed by ATP binding (n = 18 to 34), enzyme binding (n = 11 to 20), and ATP hydrolysis activity (n = 10 to 20).

**Figure 6:**
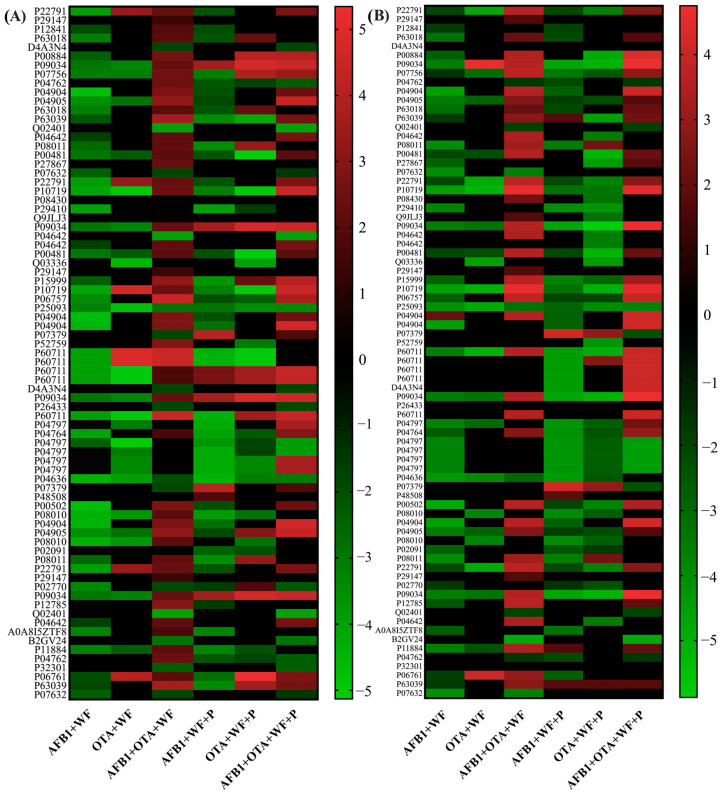
Heatmap representation on the expression of DEPs involved in the main biological processes after AFB1, OTA, and the combination (AFB1 + OTA) exposure in presence of FW or FW + P in male (**A**) and female (**B**) rats compared to control. The red-to-green gradient represents the logarithmic fold change value for upregulation (Log2FC > 0) and downregulation (Log2FC < 0), respectively. Black box is log2FC = 0. *p* < 0.05 significantly different from the mycotoxin groups.

To deepen these results, a heatmap was generated from the proteomic data to visually represent the general changes in protein expression following exposure to AFB1, OTA, and their combination (AFB1 + OTA) in the presence of FW or FW + P in both male and female rats compared to control feed (Figure 6). The heatmap displays downregulated proteins (green) and upregulated proteins (red), providing a clear overview of the proteomic response to mycotoxin exposure across the different conditions. The detailed list of DEPs altered in BPs is included in Supplementary Material Table S1 for male and Table S2 for female.

Steady outcomes were perceived in both sexes, revealing matching trends in protein expression. When exposing rats to each mycotoxin separately supplemented with FW or FW + P, the preponderant part of proteins displayed a moderate downregulation (LogFC < −2), particularly in the AFB1 group, hinting at the positive action of bioactive compounds against the toxin. However, occasionally with OTA, a few proteins were upregulated, especially in males and with both ingredients. Differently, when rats were exposed to the mycotoxin mixture, the expression profile outlined a significant upregulation (LogFC > 2), particularly in the combined group (AFB1 + OTA + FW + P). This condition exhibited a potential synergistic consequence of the two toxins, where the simultaneous exposure may exacerbate the biological response compared to a single one. In line with that, numerous investigations have previously reported an AFB1 and OTA additive effect in vivo and in vitro, emphasizing the potential risk of their co-occurrence [34]. A recent metabolomic study, for instance, reported a synergistic effect of AFM1 and OTA in mice livers, displaying the alteration of metabolites related to oxidative stress [35].

### 2.3. Metabolic Pathways Analysis

**Figure 7:**
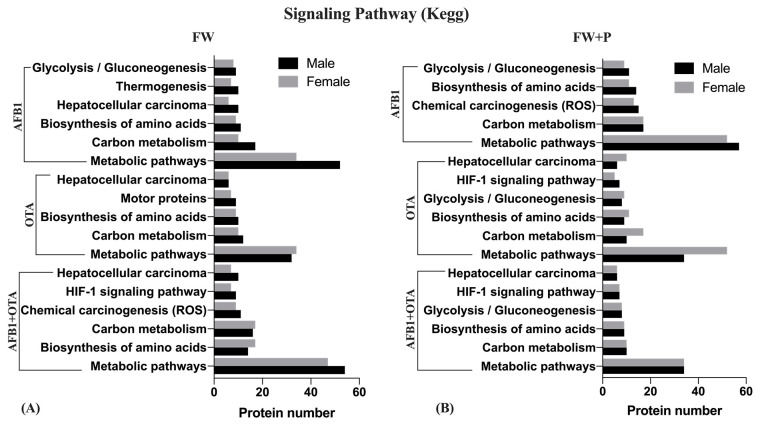
KEGG pathway visualization of significant signaling pathways in rats exposed to mycotoxins in combination with fermented whey (FW) (**A**) or fermented whey + pumpkin (FW + P) (**B**) feed related to the number of proteins involved compared with the exposure without functional ingredients.

Understanding the mechanism of action of pathways involved in the primary functioning of the liver has helped to clarify the metabolic alterations that occur in the presence of AFB1 and OTA and, notably, verify the beneficial role of the functional ingredients. For this purpose, the KEGG visualization tool related to DEPs in this study allowed the identification of the main processes altered in rats exposed to feed tainted with mycotoxins and combined with FW or FW + P, reporting that the most significant signaling pathways affected were predominantly linked to metabolic responses (Figure 7). In fact, these routes showed the highest number of modified features, higher in males exposed to FW and AFB1 alone (*n* = 54) or in combination with OTA (*n* = 52) whereas, in females, they were lower with single mycotoxins (*n* = 34) than combined (*n* = 52). In the presence of pumpkin, the situation was reversed between the genders.

However, it is well known that the liver is the largest metabolic organ which plays a specific role in digestion, metabolism, absorption, and transport of nutrients, biodegradation of toxic compounds, and processing of various hormones and cytokines secreted by the viscera [36]. Moreover, it is essential for the biosynthesis of amino acids (AAs) that serve as the building blocks for several key proteins as well as being the center of glucose metabolism and fatty acid diverting [37]. In the present investigation, carbon metabolism emerged among the most commonly altered pathways (Figure 7), along with biosynthesis of AA, suggesting consequent disruptions in energy production besides cellular processes. Indeed, one-carbon metabolic pathways aim to activate serine metabolism to glycine, the glycine cleavage system (GCS), and the metabolism of choline and other amino acids. For that reason, recent studies indicated that cancer cells may modify or become increasingly dependent on these pathways in order to maintain the supply of carbon units which are necessary for their proliferation [38]. Additionally, chemical carcinogenesis of ROS and hepatocellular carcinoma were also impacted (*n* > 10), particularly relevant given the liver’s central role in metabolizing AFB1 and OTA.

Focusing once more on the overall expression of DEPs, the heatmap revealed a distinct pattern in protein expression across the multiple groups. The detailed list of DEPs altered in MPs is included in Supplementary Material Table S3 for male and Table S4 for femal.

**Figure 8:**
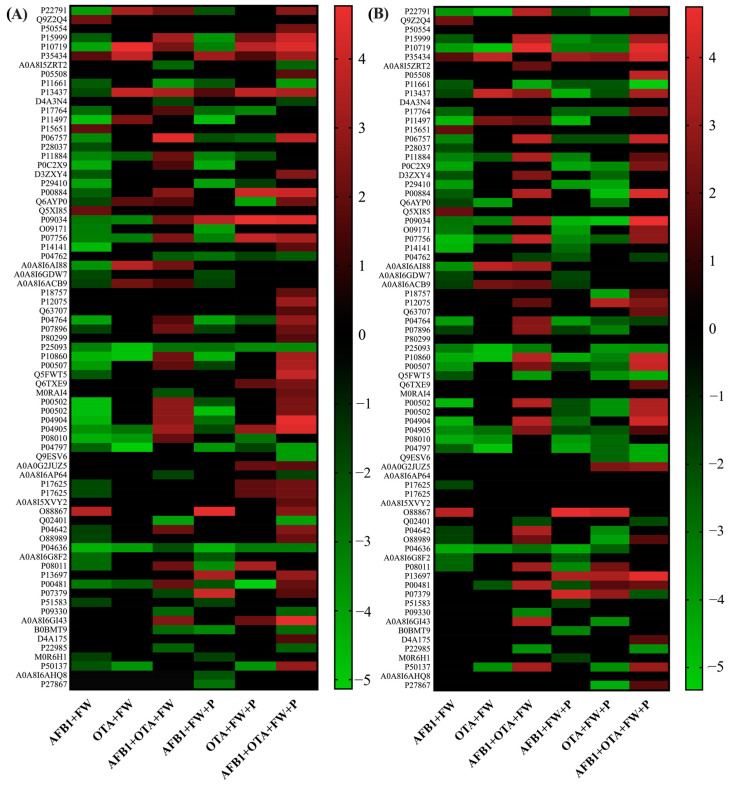
Heatmap representation of the expression of DEPs involved in the main signaling pathways after AFB1, OTA, and the combination (AFB1 + OTA) exposure in presence of FW or FW + P in male (**A**) and female (**B**) rat livers compared with the expression after exposure to mycotoxins without functional ingredients. The red-to-green gradient represents the logarithmic fold change value for upregulation (LogFC > 0) and downregulation (LogFC < 0), respectively. Black box is log2FC = 0. *p* < 0.05 significantly different from the mycotoxin’s groups.

In this case, when exposing rats to AFB1 + FW and AFB1 + FW + P, an extend downregulation can be observed compared with AFB1 (Log < −1.6). Very small differences between sexes were found in the downregulation trend observed with AFB1 exposure with the inclusion of functional ingredients compared with the mycotoxins only. With OTA, the downregulation trend can be observed more clearly when adding FW + P, especially in females (Figure 8). Nonetheless, in the latter, an upregulation of certain specimens is also displayed, reporting higher values in females (LogFC > 2.0) than in males (LogFC > 1.7). Conversely, when both AFB1 and OTA were administered together, a clear upregulation of proteins was observed, suggesting once again a synergistic effect between the two toxins. Nevertheless, the increase in protein expression in response to combined toxin exposure was slightly more pronounced in females (LogFC up to 4.7) (Figure 8A) than in males (LogFC up to 4.5) (Figure 8B), further supporting the hypothesis of a stronger synergistic effect in females. Thus, the contribution of FW or FW + P in the diet modulated the toxic effects of AFB1 and OTA when they were administered singularly, highlighting the potential for combined exposures to exert stronger effects than individual toxins.

**Figure 9:**
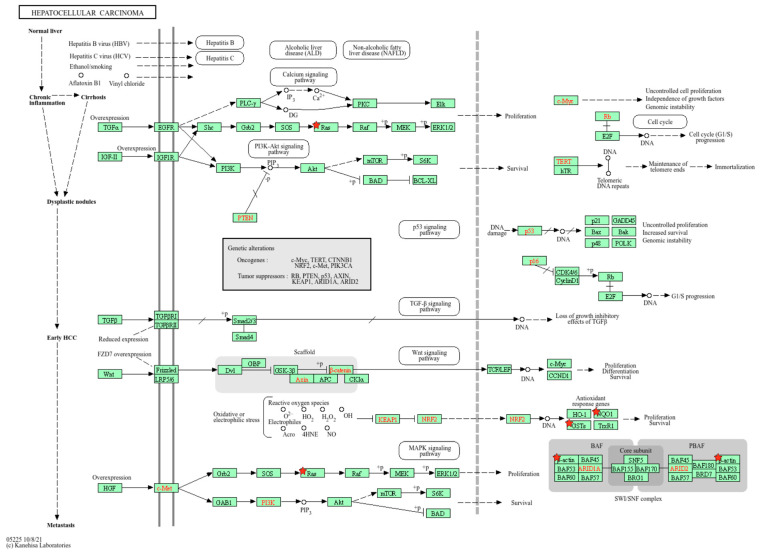
KEGG pathway visualization showing key molecular events involved in the development of hepatocellular carcinoma (HCC). The diagram highlights the critical signaling pathways, including those related to cell cycle regulation, apoptosis, and metabolic alterations, which contribute to the initiation and progression of liver cancer. Red stars indicate DEPs found in this study after AFB1 and OTA exposure and bioactive ingredients.

Remarkably, several proteins identified through the proteomic analysis were linked to the hepatocellular carcinoma (HCC) pathway, a key area of concern following exposure to mycotoxins. In fact, HCC is a primary form of liver cancer which often results from chronic exposure to various toxic agents and is the sixth most common malignancy worldwide [39]. In this study, several proteins involved in liver function and cancer development were significantly affected by AFB1 and OTA exposure (Figure 9).

Among them, important members of the actin family such as beta actin (Actb), beta-actin 2 (Actbl2), actin gamma 1–1 (Actg1l1), and actin gamma 1 (Actg1) often dysregulated in cancer were downregulated under exposure to individual mycotoxin and bioactive ingredients, hinting at their helpful role. In fact, these actin monomers are fundamental for cytoskeletal polymerization and integrity and are directly implicated in the maintenance of assembly and turnover of diverse cellular processes [40] and were upregulated in male and female rats after exposure to mycotoxins individually. Among them, Actb, Actg1, and Actin 5 (Act5) were strongly upregulated after single administration (LogFC > 14).

Additionally, antioxidant response proteins belonging to the glutathione S-transferase (GST) and NAD(P)H quinone oxidoreductase 1 (NQO) families were significantly downregulated in male and female rats, with LogFC < −2.6 for FW + AFB1 and LogFC < 1.8 with both functional ingredients. On the contrary, in the combined exposure (AFB1 + OTA), expression was increased (LogFC > 2), as was the case in the single mycotoxin administration of AFB1 (LogFC > 10), highlighting a shift in cellular signaling that could favor tumorigenesis. In fact, GST is a key regulator of phase II enzymes which protect cells from oxidative stress in cancer [41], and herein, six types of GST-related proteins were broadly altered: GSTa1, GSTa2, GSTa3, GSTm1, GSTm2, and MGST1. Likewise, the upregulation of NPQ1, as in this case, has been associated with human liver injury [42]. Overall, the expression of the abovementioned proteins, particularly in combination with functional ingredients, could serve as potential biomarkers for liver carcinogenesis through the identification of important targets for therapeutic intervention.

The present study highlights significant sex-specific differences in the hepatic response to mycotoxins (AFB1 and OTA) and their mitigation by bioactive compounds such as FW and P. These differences were evident in the number of differentially expressed proteins (DEPs), the biological processes (BPs) affected, and the pathways modulated under various experimental conditions.

Female rats consistently exhibited a higher number of DEPs compared to males across all experimental groups. This disparity suggests a greater sensitivity of females to mycotoxin-induced hepatic changes. For instance, in response to AFB1 exposure, females exhibited 134 DEPs compared to 95 in males, while the combined AFB1 + OTA exposure amplified this effect further (140 DEPs in females vs. 81 in males). These findings align with previous reports suggesting that sex hormones may influence xenobiotic metabolism and the oxidative stress response, potentially rendering females more vulnerable to hepatotoxic effects [43,44]. The observed downregulation of key antioxidant enzymes such as Gpx1, Sod1, and Cat in females further corroborates this hypothesis, as it indicates a diminished capacity to counteract oxidative stress. In contrast, males displayed a more robust response to xenobiotic stimuli, suggesting a higher activation of detoxification pathways mediated by phase I and phase II enzymes.

Supplementation with FW or FW + P exhibited protective effects in both sexes, though the mechanisms and extent of mitigation differed. The response to xenobiotic stimuli emerged as a predominant biological process in males (18–20 proteins involved) compared to females (9–17 proteins), reflecting a sex-dependent variation in detoxification capacity. Conversely, females demonstrated a greater modulation of oxidative-stress-related pathways and metabolic processes, including amino acid biosynthesis and the urea cycle.

Interestingly, the combination of FW and P enhanced the mitigation effects, with a higher number of DEPs observed in both sexes compared to FW alone. For instance, FW + P supplementation in AFB1-exposed females resulted in 137 DEPs, compared to 127 in males. These findings suggest a synergistic effect of FW and P in modulating hepatic responses to mycotoxins, particularly in pathways related to cellular repair and antioxidant defense.

Combined exposure to AFB1 and OTA exacerbated the hepatotoxic effects, particularly in females, as evidenced by a more pronounced upregulation of proteins (LogFC up to 4.7 in females vs. 4.5 in males). This suggests a synergistic interaction between the two mycotoxins that overwhelms the hepatic defense mechanisms, especially in females. The adaptive response observed in females, characterized by an increase in structural chromatin proteins and metabolic enzymes, may represent an effort to counteract the heightened toxic burden. However, this response appears to be less effective compared to the more stable protein expression profiles observed in males.

## 3. Conclusions

Proteomics studies of Wistar rats exposed to AFB1 and OTA with the addition of FW and P have highlighted their ability to counteract the negative effects of mycotoxins on hepatic responses, particularly in detoxification and development processes. Moreover, metabolic alterations induced by these toxins evidenced a significant variation in carbon metabolism and biosynthesis of AA, included in the liver’s main functions. Interestingly, important biomarkers implicated in HCC were positively modulated by functional ingredients in both males and in females, but only with mycotoxins individually.

Based on these findings, the presence of FW or FW + P as functional ingredients in food may play a significant role in modulating toxic responses of mycotoxins, though further analysis is needed to fully elucidate the protective mechanisms.

## 4. Material and Methods

### 4.1. Reagents

For feed preparation, wheat flour, water, salt (NaCl), and sugar (sucrose) were acquired from a commercial market in Valencia, Spain. Aspergillus flavus ITEM 8111 was purchased from the Agro-Food Microbial Culture Collection of the Institute of Sciences and Food Production (ISPA, Bari, Italy) whereas Aspergillus steynii 20,510 was obtained from Spanish Type Culture Collection, CECT, Science Park of the University of Valencia (Paterna, Valencia, Spain). Goat milk whey coagulated by commercial rennet (starter culture R-604) was purchased from the ALCLIPOR society, S.A.L. (Benassal, Spain) while pumpkin used in this study was purchased from a supermarket (Valencia, Spain). It was peeled, the seeds removed, cut, and freeze-dried to then grind and obtain a homogeneous powder.

For protein precipitation, extraction, and digestion, ethanol was supplied by Sigma-Aldrich (St. Louis, USA), and dithiothreitol (DTT) with a purity of 99%, Trizma^®^ hydrochloride, Tris-HCl with a purity of 99%, and trypsin were purchased from Sigma-Aldrich (St. Louis, MO, USA). Thiourea, purchased from Thermo Fisher Scientific (Kandel, Germany), and urea obtained from FEROSA (Barcelona, Spain) were used to prepare the lysis buffer used in protein digestion. Furthermore, iodoacetamide (IAA) with a purity of 98% was obtained from ACROS OrganicsTM, Thermo Fisher Scientific (Princeton, NJ, USA).

Finally, for proteomics analysis, methanol was supplied by Sigma-Aldrich. Acetonitrile (AcN) LC/MS-grade OPTIMA^®^ (≥99.9% purity) was supplied by Fisher Chemical (Geel, Belgium). Formic acid (≥98%) was obtained from Sigma-Aldrich. Deionized water (<18, MΩcm resistivity) was obtained using a Milli-Q water purification system (Millipore, Bedford, MA, USA).

### 4.2. In Vivo Experimental Design

Male and female Wistar rats (weighing between 260–340 g) were obtained from the pharmacy animal facility at the University of Valencia, Spain. At the beginning of the study, rats were housed in polycarbonate cages in a windowless room with a 12 h light/dark cycle. The room conditions were carefully controlled to meet the species’ requirements, with a temperature of 22 °C and relative humidity maintained between 45–65%. To ensure sterility during the procedures, nitrile gloves and FFP3 masks were worn when handling the animals or contaminated samples. This study was approved by the Animal Care and Use Committee of the University of Valencia (2021/VSC/PEA/0112).

After seven days of acclimatation, a total of 120 Wistar rats were divided into 12 groups, each consisting of 10 rats (5 males and 5 females) for the corresponding feeds. Among them, four test groups received mycotoxins individually or in combination, four were fed FW-contaminated feed, and the other four FW-P-containing contaminated feed. For the feeds containing functional ingredients, 35 g of FW and P were added to each during the preparation. This amount represents 1% (*w*/*w*). The control group was fed uncontaminated feed. The experimental conditions related to mycotoxin doses and their respective standard deviations are reported by [45].

The doses of aflatoxin B1 (AFB1) and ochratoxin A (OTA) used in the study were calculated based on the levels in the contaminated feed and the rats’ daily intake: AFB1 dose varies from 176 to 387 µg/kg body weight per day, depending on the experimental group and sex of the rats. The dose of OTA ranged from 162 to 552 µg/kg body weight per day, with females generally receiving higher doses than males due to differences in feed intake relative to body weight. These doses were derived from feed containing AFB1 and OTA at concentrations of approximately 4.3–5.2 µg/g for AFB1 and 5.4–8.8 µg/g for OTA and were adjusted for body weight and feed consumption to reflect realistic exposure scenarios.

After 28 days, rats were sacrificed following isoflurane inhalation and organs were stored at −80 °C.

### 4.3. Protein Extraction, Reduction, Alkylation, and Digestion

Protein extraction was initiated using 50 mg of liver tissue which was homogenized in MilliQ-H_2_O using an Ultra Turrax (IKA T10 standard). Afterwards, proteins were precipitated twice by adding 2 mL of cold ethanol to each sample, bringing the final volume to 2.5 mL. Samples were then centrifuged at 4.000 rpm, 4 °C for 15 min, the supernatant was discarded, and the pellets were resuspended in 500 μL of H_2_O. Protein concentration was determined using a NeoDot UV/Vis Nano Spectrometer(ref) in order to standardize the concentration to 1 mg/mL to start the digestion. Subsequently, samples were resolved in 200 μL of lysis buffer (8 M urea/2 M thiourea/50 mM Tris-HCl) and underwent reduction and alkylation by adding solutions of DTT and IAA at a concentration of 200 mM and pH 7.8, prepared with MilliQ-H_2_O and 0.4 M Tris stock buffer (pH 7.8, Tris base/MilliQ-H_2_O). To break disulfide bonds, samples were incubated with 5 μL of DTT 200 mM for 1 h at 60 °C in a ThermoMixer C (Eppendorf). Samples were then incubated for 30 min at 37 °C to alkylate protein cysteine residues with 20 μL of IAA. Finally, trypsin enzyme (1 mg/mL) was added to start peptide digestion which was carried out overnight at 37 °C. After that, the reaction was stopped by adding acetic acid 5% (pH 5) and filtered prior to LC-MS/MS-Q-TOF injection.

### 4.4. Identification and Quantification of Proteins Through LC-MS/MS-Q-TOF

Two technical replicates of each biological sample (50 μg/mL) were injected into an LC system (Agilent 1200 LC) coupled to a triple quadrupole time-of-flight (Q-TOF) mass spectrometry (Agilent 6540 UHD) system using a C18 RP AdvanceBio capillary column for 2.7 μm, 120 Å, 2.1 × 150 mm peptide mapping. The method previously developed by [28] was followed. Briefly, a nonlinear gradient of 40 min at a flow rate of 0.2 mL/min was utilized. Two different phases were used in the process: phase A (H_2_O in 0.1% formic acid) and phase B (acetonitrile in 0.1% formic acid). The elution gradient starts with 3% phase B for 1 min and increases to 40% at 21 min. In the next 3 min, it reaches 95% and was maintained during 1 min; Afterwards, it decreases to 3% for 6 min and maintained in the last 8 min. The experimental conditions were repeated three times independently.

### 4.5. Statistical Analysis and Bioinformatics

The software Spectrum Mill MS Proteomics Workbench Package Rev B.06.00.201 (Agilent Technologies) was used to process the chromatographic spectra. This software is capable of analyzing data from high-quality spectra, reducing false positives, and identifying proteins and peptides by matching them with the UniProt database. Entities were then sorted by their frequency of occurrence across all replicates within each experimental group following the MS/MS parameters previously retrieved and verified by [46]. Afterwards, the identified proteins were statistically filtered by using Mass Profiler Professional (MPP) software v15.0 (Agilent Technologies) and differences between the experimental mycotoxin and the control group were assessed using an unpaired *t*-test with Benjamini–Hochberg adjustment. Results with a FC ≥ 0.7 and a *p*-value < 0.05 were considered statistically significant and checked for the bioinformatics analysis, including the features which corresponded to the UniProt accession codes for *Rattus norvegicus*. Finally, the BPs, MFs, and metabolic pathways associated with these proteins were explored using the Database for Annotation, Visualization, and Integrated Discovery (DAVID). Graphical representations of the data were created with GraphPad Prism software version 8.0.0 (San Diego, CA, USA). The Venn diagram for DEPs was generated using the Venny 2.1 interactive tool [47].

## Data Availability

The original contributions presented in this study are included in this article and Supplementary Material. Further inquiries can be directed to the corresponding authors.

## Supplementary Materials

The following are available online at https://www.mdpi.com/article/10.3390/toxins17010029/s1, Table S1: DEPs obtained through statistical analysis comparing male rats exposed to mycotoxins and FW (AFB1+FW, OTA+FW, AFB1+OTA+FW) or FW+P (AFB1+FW+P, OTA+FW+P, AFB1+OTA+FW+P) versus the corresponding mycotoxin (AFB1, OTA, AFB1+OTA) related to biological processes. Values represent the Log2FC. Table S2: DEPs obtained through statistical analysis comparing female rats exposed to mycotoxins and FW (AFB1+FW, OTA+FW, AFB1+OTA+FW) or FW+P (AFB1+FW+P, OTA+FW+P, AFB1+OTA+FW+P) versus the corresponding mycotoxin (AFB1, OTA, AFB1+OTA) related to biological processes. Values represent the Log2FC. Table S3: DEPs obtained through statistical analysis comparing male rats exposed to mycotoxins and FW (AFB1+FW, OTA+FW, AFB1+OTA+FW) or FW+P (AFB1+FW+P, OTA+FW+P, AFB1+OTA+FW+P) versus the corresponding mycotoxin (AFB1, OTA, AFB1+OTA) related to metabolic pathways. Values represent the Log2FC. Table S4: DEPs obtained through statistical analysis comparing male rats exposed to mycotoxins and FW (AFB1+FW, OTA+FW, AFB1+OTA+FW) or FW+P (AFB1+FW+P, OTA+FW+P, AFB1+OTA+FW+P) versus the corresponding mycotoxin (AFB1, OTA, AFB1+OTA) related to metabolic pathways. Values represent the Log2FC.
